# Accuracy and Feasibility of Optoelectronic Sensors for Weed Mapping in Wide Row Crops

**DOI:** 10.3390/s110302304

**Published:** 2011-02-24

**Authors:** Dionisio Andújar, Ángela Ribeiro, César Fernández-Quintanilla, José Dorado

**Affiliations:** 1 Institute of Agricultural Sciences, CSIC, Serrano 115B, 28006 Madrid, Spain; E-Mails: andujar@ccma.csic.es (D.A.); cesar@ccma.csic.es (C.F.-Q.); 2 Centre for Automation and Robotics, CSIC-UPM, 28500 Arganda del Rey, Madrid, Spain; E-Mail: angela@iai.csic.es (A.R.)

**Keywords:** weed detection, ground-based mapping system, sampling resolution, site-specific weed management

## Abstract

The main objectives of this study were to assess the accuracy of a ground-based weed mapping system that included optoelectronic sensors for weed detection, and to determine the sampling resolution required for accurate weed maps in maize crops. The optoelectronic sensors were located in the inter-row area of maize to distinguish weeds against soil background. The system was evaluated in three maize fields in the early spring. System verification was performed with highly reliable data from digital images obtained in a regular 12 m × 12 m grid throughout the three fields. The comparison in all these sample points showed a good relationship (83% agreement on average) between the data of weed presence/absence obtained from the optoelectronic mapping system and the values derived from image processing software (“ground truth”). Regarding the optimization of sampling resolution, the comparison between the detailed maps (all crop rows with sensors separated 0.75 m) with maps obtained with various simulated distances between sensors (from 1.5 m to 6.0 m) indicated that a 4.5 m distance (equivalent to one in six crop rows) would be acceptable to construct accurate weed maps. This spatial resolution makes the system cheap and robust enough to generate maps of inter-row weeds.

## 1. Introduction

The main challenge in weed control under Precision Agriculture is to know weed distribution throughout the field so that the site-specific weed management can be performed. Usually, weed mapping operations are expensive and can make Precision Agriculture economically unfeasible, therefore the design and development of effective procedures for weed mapping are essential. Weed mapping in agricultural crops can be performed by various procedures, including manual surveys, sensors located on ground vehicles or remote sensing. Although discrete manual sampling methods have been used in the past for research purposes, they are too time consuming to be acceptable in commercial use [1,2]. Continuous mapping systems based on visual assessments of weed infestations conducted from all-terrain vehicles or from combine harvesters provide a simple, cost-effective solution for mapping patchy-distributed weeds in commercial fields [2–4]. However, these systems rely heavily on human perception and have various other limitations.

Remote sensing has been commonly considered as an effective technique for weed patch delineation [5–7]. Nevertheless, the use of satellite and airborne methods is strongly dependent on sky cloudiness. This is a major limitation due to the relatively short time window available for weed detection and subsequent control actions (e.g., herbicide spraying). Considering that additional time is required for data processing, this may cause undesirable delays in herbicide application.

Ground-based, machine-mounted sensors offer numerous advantages for practical weed mapping. These sensors are relatively independent of the environmental conditions, they can be used on real-time applications or shortly before herbicide treatment and, depending on the type, they potentially may discriminate low weed densities. Various ground-based sensing systems have been used for weed mapping. Most of these studies have used machine vision techniques to detect and identify plant species (either crops or weeds) based on their shape, texture, colour and location based features individually or jointly [8–11]. As remote sensing, machine vision sensing systems essentially require image acquisition and image processing techniques [12], which usually are computationally expensive. Another challenge in outdoor machine vision weed sensing is the variable lighting conditions when using conventional digital video cameras, an aspect especially important when it comes to real-time operations [13]. Recent works have described automatic methods for mapping weeds in the field using a digital video camera for continuous image capture along the crop seedline from a moving vehicle equipped with a GPS receiver [14,15]. A manual validation of the accuracy of the image processing method conducted on a random sample of video frames indicated that 74% of the weeds present were correctly identified [14]. However, the practical feasibility of computer vision equipment in ground-based agricultural field operations continues to be a challenge for large-scale weed mapping. In addition, this equipment is dependent on crop features and needs to be adapted to the crop and weed type.

In certain scenarios (e.g., crops in wide row spacings, fallow, crop preemergence), all green plants are weeds. Under these conditions, the development of different vision systems to detect weed plants in real-time for site-specific spraying of infested areas has been proposed by several researchers [16,17]. These systems can be based on optoelectronic sensors and used for the discrimination between vegetation and soil from their reflection spectra. Shearer and Jones [18] used this type of sensor to detect weed growth. Biller [19] used these sensors to detect weeds between rows of maize, in order to perform a site-specific weed control. The results of this study showed a significant saving in herbicides, reducing the spraying fluid used by 30 to 70% in comparison with conventional application of the entire field. Although optoelectronic sensors are not able to distinguish between weeds and crops, this does not represent a problem if the sensor is operated only in the inter-row area. The non-specific information generated by this type of sensor may be useful for indicating areas at high risk of weed infestation. High- and low-risk areas can be managed differentially either with herbicides or with other control techniques. Fast and reliable weed mapping tools are also needed to characterize and study weed populations in research studies conducted in large commercial fields. Recently, Sui *et al.* [20] used this approach to identify the relationships among airborne multi-spectral imagery and ground truth data of weed intensity obtained with optoelectronic sensors.

The high cost of weed detection technologies is a major deterrent for their commercial introduction. Thus, a high spatial resolution, real-time weed detection system seems to be the solution for site-specific weed management. In this regard, the spatial resolution at which weed mapping is conducted is likely to be a major factor in determining its cost effectiveness [21,22]. Berge *et al*. [23] showed that mapping errors increased gradually by increasing the distance between image samples and size of control area, *i.e.*, with spatial resolution. Since an increasing resolution may have an important associated cost, it is relevant to find out the maximum sensor distance that may provide a reliable description of weed distribution.

The objectives of this work were: (i) to evaluate the accuracy and performance of a ground-based weed mapping system involving optoelectronic sensors for weed detection in maize fields; and (ii) to assess the influence of distance between sensors on mapping errors.

## 2. Experimental Section

### 2.1. Description of the Optoelectronic Mapping System

**Scheme 1.**
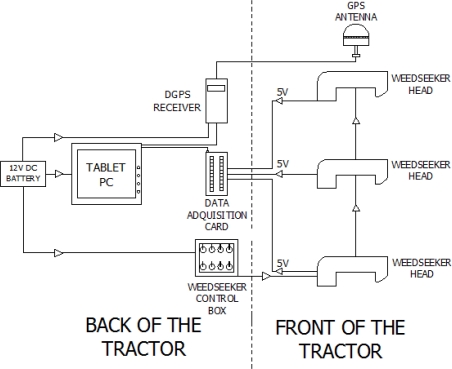
Ground-based weed mapping system with the components for weed detection and weed geo-positioning in the front of the tractor, and with the components for data acquisition and processing at the back of the tractor.

A ground-based weed mapping system combining optoelectronic sensors with location information was used to map weed distribution within maize fields. The mapping system consisted of three major components: plant detection sensors, DGPS receiver and devices for data acquisition and processing (Scheme 1).

**Scheme 2.**
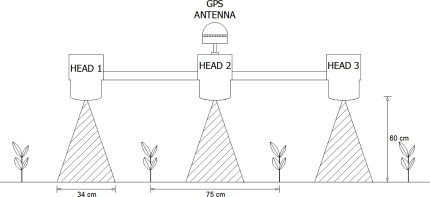
Location of the optoelectronic sensors on the crop rows as well as the position of the DGPS antenna and the sensor viewing area (0.34 m × 0.02 m) located in the middle of each inter-row area.

For plant detection, the system employed the optical technology included in the WeedSeeker^®^ sensor (NTech Industries Inc., Ukiah, CA, USA), which is an active optical sensor with its own light source and therefore is usable at any time, day or night. The sensor distinguishes green plants from bare ground by their different light reflection in the red and near infrared bands. Three optoelectronic sensors were mounted on the front of a tractor (John Deere 1140) at 0.75 m intervals and at 0.60 m height above ground level (Scheme 2). Hence, the system was able to explore three rows of maize, viewing 0.34 m × 0.02 m (perpendicular × parallel to the travel direction, respectively) strips in the middle of each inter-row area. The output signal sent continuously by the sensor, *i.e.*, 5 V or 0 V when green plants were or were not detected, respectively, was redirected to a data acquisition board with three 5 m long cables. Optical calibration of the sensors was performed from the WeedSeeker^®^ controller panel on a bare (weed-free) area of ground in each field and their sensitivity was adjusted in order to detect weeds covering about 15% of the surface. This value was consistent with published data on weed cover that produce a significant reduction in maize yields [24]. Preliminary tests conducted with various sensitivity adjustments of the sensors indicated that a medium sensitivity (switch in position 6) resulted in a threshold of 15% weed cover generating a 5 V output signal. This means that all areas with weed cover below this threshold were not detected.

Weed location information was obtained from a differential GPS (DGPS) receiver Hemisphere Crescent R130 (Hemisphere GPS, Calgary, AB, Canada), with Omnistar correction signal capable of sub-meter accuracy (about 0.4 m), working with a 5 Hz frequency. The DGPS antenna was located on top of the central optoelectronic sensor (Scheme 2). Geo-positioning of the data obtained with the two other sensors was corrected during post-processing. With the tractor at constant velocity of 5 km/h, the working/analysing capacity of the system was about 1 ha/h.

The data acquisition device was a USB-based data acquisition module Labjack U12 (LabJack Corporation, Lakewood, CO, USA) which had 20 digital I/O channels that could be individually configured as input or output and it was connected through the USB connector to a processing device, an Itronix Duo-Touch™ Tablet PC (1.1 GHz processor, 8.4” TFT SVGA outdoor transmissive display and Windows XP Tablet PC applications). The DGPS receiver signal was collected by the Tablet PC through a USB port. In addition, the analog signals from the three optoelectronic sensors were input to the Tablet PC from the data acquisition board with a sampling frequency of 100 Hz. Therefore, the distance between the sensor measurements was limited by the frequency of the DGPS receiver, *i.e*., about 0.3 m. The data capture and user-system interface software used was developed for the weed mapping application. Post-processing of the data was conducted with ArcGis^®^ 9.2 software (ESRI, Redlands, CA, USA).

The WeedSeeker^®^ controller panel, the DGPS receiver, the data acquisition board and the Tablet PC were placed on a toolbar-mounted unit located in the back of the tractor (Scheme 1), in front of the operator. All these instruments were directly powered by the 12 V battery of the tractor.

### 2.2. Study Sites

Field experiments were conducted in La Poveda Research Farm (Arganda, Central Spain). The climate of the site is Mediterranean Continental with cold winters, hot summers and limited precipitation of about 400 mm. Three maize fields have been used in this study, following normal agricultural practices in this culture. Thus, the three fields were planted in early April with 0.75 m row spacing and a population of 90,000 plants/ha, and sprinkler irrigated, with the first irrigation applied 1 month after planting and weekly irrigations thereafter. Weeds were assessed in May when maize was at the stage 14 to 16 of BBCH scale [25]. Field A (2.5 ha) had been cropped with maize continuously in the five years before this study and was heavily infested with *Sorghum halepense* (L.) Pers., *Datura ferox* L. and *Xanthium strumarium* L., with very sparse weed free areas. In contrast, field B (3.0 ha) and field C (1.7 ha), which had rotated with winter barley, showed less infestation of weeds with some patches of *S. halepense*, *X. strumarium*, *Datura stramonium* L. and *D. ferox*. Herbicide treatments, fertilization and other agricultural operations were also those commonly used in maize crops.

### 2.3. Analysis of the Accuracy of the Optoelectronic Mapping System

In order to verify the system, digital images were obtained on the same day in a regular 12 m × 12 m grid. Using a discrete area sampling, *i.e.*, weed assessment within a quadrat on a grid basis, is the standard practice to describe the spatial distribution of weeds within a field [1,26]. A total of 160 and 112 georeferenced points were obtained in field A and field C, respectively. In field B, only one third of the surface was sampled, obtaining a total of 70 images. The position of the sampling points was acquired with the above indicated DGPS receiver by placing the antenna manually on the midpoint of each quadrat. A Nikon digital camera D70 equipped with 18–70 mm AF-S DX Nikkor lens was used to capture the digital images. The camera incorporates a 6.1-effective megapixel DX Format CCD image sensor that produces 3,008 × 2,000-pixel images, sufficient to show clearly any green objects in the image. The images were taken on the inter-row area, each image covering 0.28 m^2^ (0.7 m × 0.4 m). During image collection, the camera was handheld at about 1.30 m height and direct sunlight was avoided on image area with a white umbrella. Weed cover present in each image was assessed independently by image processing software developed by the authors [11]. Then, percentage cover values were transformed to a 0/1 rating system, with 0 corresponding to values below the threshold of 15%, *i.e.*, absence of weeds, and with 1 corresponding to values above 15%, *i.e*., presence of weeds. This threshold has been reported previously in studies of weed-crop interference as the weed cover threshold from which there was a significant decrease in maize yields [24]. These weed cover values were considered as the “ground truth”.

Verification of the data obtained with the optoelectronic mapping system was performed by comparing the agreement on information of weed presence/absence at points where geo-referenced digital images were obtained. For this purpose, contingency tables [27] were created to analyse the relationship (percentage of agreement) between the data obtained with the optoelectronic mapping system and the information from the digital images. Since weed populations in agricultural fields usually occur in patches [28], it is widely accepted that weed densities within fields are generally autocorrelated: if density in a specific area (*i.e.*, image of 0.28 m^2^) is high it is likely that the densities in the neighbouring areas will also be high; and if the sampled quadrat is free of weeds it is likely that the surrounding areas will also be weed free. Consequently, the variability within each quadrat would be small compared with the variation between weedy and weed-free zones [26]. In order to assess the variability within the images, each quadrat was divided into 20 segments of 0.70 m × 0.02 m (perpendicular × parallel to the travel direction, respectively), *i.e*., the same size than the narrow side explored by the sensor. A Kolmogorov-Smirnov test of goodness-of-fit of a uniform distribution [27] was performed to check data variability in the 20 segments within each image. The analysis of all images showed a uniform distribution of weeds within the quadrat in most cases with only 11.8% showing non-uniformity. Cases with non-uniformity within the quadrat were mainly found (about two thirds of the 11.8%) in images with densities close to the defined threshold (15%). Images with a low or high weed density showed a better fit to the uniform distribution. Considering the aggregate distribution of weed populations, it can be assumed the validity of reference system since sampling points of 0.28 m^2^ coincide in most cases (45% quadrats) outside the patch, *i.e*., uniform density of bare soil, or within a patch (27% quadrats), *i.e*., uniform density of weeds. Relatively few cases (28% quadrats) coincide at the edge of a patch, where the densities are close to the threshold of 15% (±5%) and the distribution of weeds within the image would not be uniform.

### 2.4. Assessment of the Sampling Resolution

The influence of distance between sensors (sampling resolution) on mapping errors was assessed by comparison of the most detailed map, obtained by monitoring all the crop rows with the sensors separated 0.75 m, with maps obtained by progressively eliminating sampling units (0.75 m wide strips) from the original detailed map. In spite of the fact that data sets are not independent, we consider more appropriate to use as reference the richer data set obtained with the sensors separated 0.75 m (*i.e.*, a total of 94,439, 115,625 and 67,464 points in field A, B and C, respectively) than the more limited set coming from geo-referenced image assessments. In addition, previous studies have shown high errors in the interpolate weed maps when the sampling grid was over 8 m × 8 m [26]. Seven distances between sensors were simulated: 1.5, 2.25, 3.0, 3.75, 4.5, 5.25 and 6.0 m, corresponding to the elimination of one to seven sampling rows. The estimation of weed cover at unsampled locations across the field was done by the geostatistical interpolation technique kriging, which is based on available data from sampled locations and on semivariogram model parameters. This interpolation technique is widely used to describe weed distribution data [2,29,30]. Due to the characteristics of the data (0/1), indicator kriging was used to construct weed maps. Kriging was conducted using ArcGis^®^ 9.2 (ESRI).

Weed mapping was performed by integrating the weed presence/absence values recorded from optoelectronic sensors with their geographical co-ordinates. Spatial variability of weeds was described using the empirical semivariogram, which characterized the average degree of similarity between weed cover values as a function of separation distance and direction [31]. Within each field, spatial correlation between data points was analyzed using the semivariance statistic [32]:

(1) $$\gamma_{h}=\frac{1}{{2N}_{h}}\sum {\left(z_{i+h}-z_{i}\right)}^{2}$$

where *γ_h_* is the empirical semivariance for the distance *h*, *N_h_* is the number of pairs of points separated by the distance *h*, and *z_i_* is the weed cover at location *i*. All pairs of point separated by distance h were used to calculate the empirical semivariogram. The pattern of anisotropy (directional influences) was considered including two directions: 0 degrees corresponding to the direction parallel to the crop row, and 90 degrees for the direction perpendicular to the crop row. Due to the presence of an anisotropic effect in the north-south direction (parallel to crop row), only this direction was used. An exponential model was utilized to fit the empirical semivariograms to the data with the “geostatistical analyst” procedure in ArcGis^®^ 9.2 software (ESRI).

Contingency tables [27] were used to cross-validate each of the maps obtained after gradually eliminating sampling units with the original detailed maps, comparing the frequency of points with similar information on weed presence/absence (percentage of agreement) in both set of data. Due to the probabilistic nature of the interpolated data in kriged maps, these values were transformed to a scale with three categories according to their probability: weed presence = *P* > 0.54; not predicted = 0.54 ≥ *P* ≥ 0.46; weed absence = *P* < 0.46. In addition, one of the parameters in the semivariogram models, the estimated geostatistical range (the scale over which spacing tend to be correlated), was utilized to further test these maps, similar to previous work [26].

## 3. Results and Discussion

**Figure 1.**
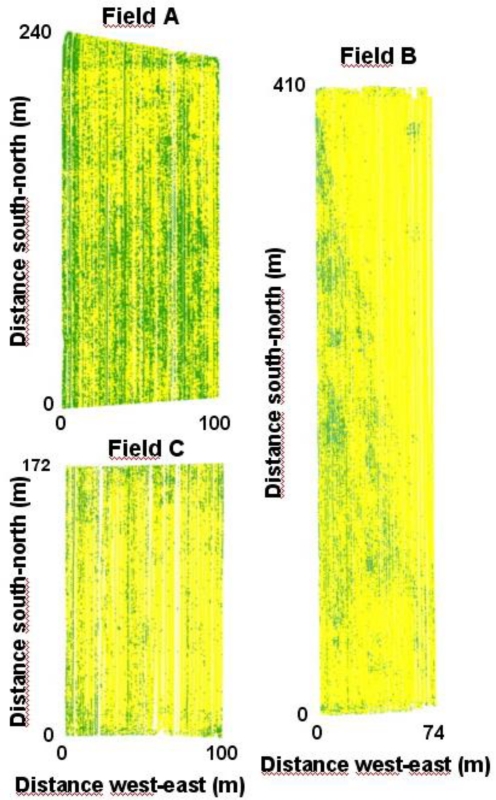
Detailed maps based upon weed sensing in all crop rows (sensors separated 0.75 m). Green points indicate presence of weeds, *i.e.*, weed coverage ≥15%. Yellow points indicate weed free areas, *i.e.*, <15% weed cover.

The original maps, obtained by sampling all the crop rows, showed two patterns of weed spatial distribution. Field A was characterized by a high weed infestation level in general (60% area infested), with only a few weed-free areas. In contrast, the two other fields were almost weed-free (about 15% area infested in both fields), with weed patches concentrated in some locations (Figure 1). This was particularly noticeable in field B, which was diagonally divided in two zones, one practically weed free whereas the other presented moderate weed infestations. This pattern was probably due to a field restructuring, with the clean zone corresponding to first year maize and the weed infested zone corresponding to the zone where maize had been grown for the last five years.

### 3.1. Accuracy of the Optoelectronic Mapping System

The comparison between the data obtained with the optoelectronic mapping system and those obtained from the digital images (“ground truth”) showed a good relationship, with 83% agreement on average between the two sets of data (Table 1). Most of the errors (about 62%) corresponded to weedy areas not detected by the sensors and the remaining 38% of errors were clean areas wrongly assessed as weedy by the sensor. A detailed study of the errors shows that most of them are found in images with a weed density close to the defined threshold of 15% (data not shown), which could be partly explained by those cases with non-uniformity within the quadrat in this range near the threshold.

Previous studies conducted with machine-vision systems have shown promising results for ground detection of weed infestations [8,9]. However, in some cases these systems may be too complex and expensive to be used for commercial weed mapping or in conjunction with patch spraying. Our research resulted in a relatively simple and robust mapping system with a large capacity for data collection. A similar system involving optoelectronic sensors tested for weed mapping in cotton was found to be reliable and easy to use [20]. Nevertheless, the accuracy of this system was not verified with any kind of ground truth data. In our study, software processing of digital images obtained in a regular grid provided a reliable reference. Other studies have used weed cover data derived from digital images to assess weed infestation [15,33]. The number of image samples used in our verification process (70 to 160 images/field) corresponds to about 0.1% of the total number of values recorded by the optoelectronic mapping system and it was higher than the sample size used in previous studies [14]. Although the total area sampled was relatively small compared with “safe” mapping resolutions proposed by Cousens *et al*. [26] and by Backes *et al*. [34], the purpose of these samples was not to describe the spatial distribution of weeds but to verify the data obtained at the same points by the optoelectronic sensors. Our results indicated a good agreement (about 83%) between the two sets of data. These results are similar to those obtained with more sophisticated sensors [14,35,36]. Regarding the disagreement between the two sets of data, special attention should be paid to the errors due to weedy areas not detected (about 11%), as these areas would escape to weed control and thus will compete with the current crop and maintain weed populations in subsequent years.

### 3.2. Optimization of the Sampling Resolution

It was assumed that higher resolution (*i.e*., a shorter distance between sensors) should provide more accurate description of weed distribution than points farther away. With this assumption, we compared the maps obtained by interpolating the raw data (0.75 m between sensors) and the maps with progressive increase of spacing between sensors (Table 2).

The contingency table showed differences greater than 10% between the maps constructed with the raw data and the other resampled maps, regardless of the distance between sensors. These differences were higher in field A, with only a 59% agreement when the distance between sensors was 6 m. These results could be a little surprising since we were expecting more accuracy (*i.e.*, higher frequency of points with similar information on weed presence/absence) in the interpolated maps in cases where the occurrence of weeds throughout the field was higher.

The sampling resolution chosen to create a map will depend on the intended end use, for instance whether the map is for research or practical purposes. The weed mapping system evaluated in this study is intended to generate prescription maps to apply herbicides when weed densities are above a given threshold. This is a very broad target and it does not require much detail in some density ranges (very low or high weed densities). However, when densities are near the threshold it is a matter of chance whether the reading of the sensor coincides with the actual situation. This is a weakness of this system that explains the relatively low agreement rate in some cases.

**Figure 2.**
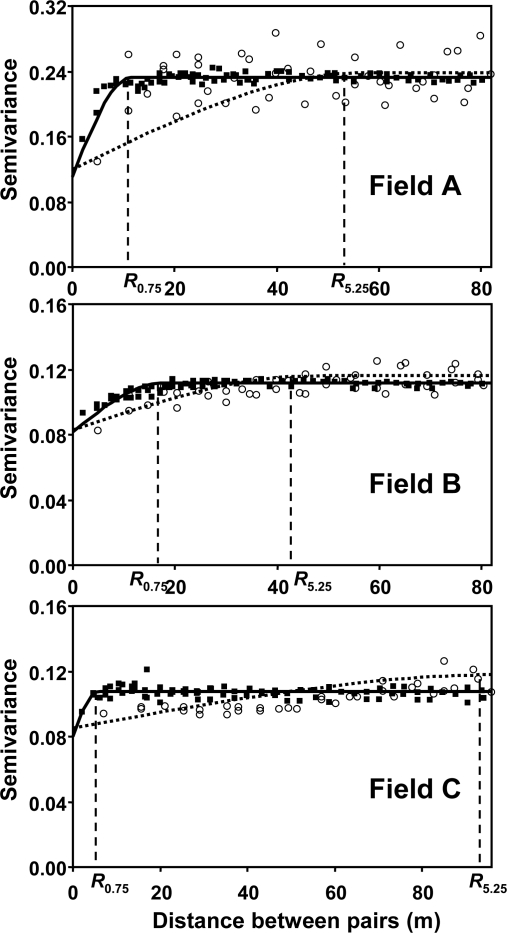
Empirical semivariograms for data obtained with sensors spaced 0.75 m (in all crop rows; square points) and with sensors spaced 5.25 m (one sensor every seven crop rows; circle points) for the north-south direction (along crop rows). Curve lines represent fitting of exponential models for sensors spaced 0.75 m (solid line) and 5.25 m (dotted line). The values of the ranges (*R*_0.75_ and *R*_5.25_ for semivariograms with sensors spaced 0.75 and 5.25 m, respectively) coincide with the points where the vertical dashed lines intersect on the X axis.

Empirical semivariograms were calculated for the north-south direction (crop row direction) due to the presence of an anisotropic (*i.e.*, directionally dependent) effect. Semivariograms showed different shapes in original maps (all crop rows sampled with sensors separated 0.75 m) and maps obtained with distances between sensors up to 6.0 m. Figure 2 shows the comparison between empirical semivariograms of the original maps and those maps with a distance between sensors of 5.25 m, in the three fields.

**Figure 3.**
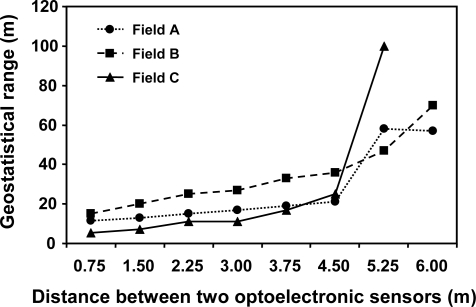
Estimates of the geostatistical range for the north-south direction (along crop rows) for weed maps constructed by the autonomous system derived from an exponential model. Three fields are compared: • Field A (2.5 ha) heavily infested by weeds; ▪ Field B (3.0 ha) and ▴ Field C (1.7 ha) with large portions of weed free areas.

Estimates of the geostatistical range (distance over which sampling spacings was correlated) increased slightly with distances between sensors from 0.75 m to 4.5 m; then, the geostatistical range showed a sharp increase at 5.25 m in all the three fields (Figure 3). The coincidence in the results of the three fields, with different patterns of spatial distribution of weeds, supports the hypothesis that it is possible to map weeds accurately using a 4.5 m distance between sensors (one sensor every six crop rows). With spacing between sensors of 5.25 m or higher, the estimates of the geostatistical range increased substantially due to the low spatial dependence in the weed community (Figures 2 and 3).

These results point to the possibility of obtaining precise weed maps with relatively coarse sampling resolution (optoelectronic sensors separated by 4.5 m, *i.e.*, one sensor every six crop rows). Consequently, this would require a low number of sensors, reducing the cost of the equipment. Wider distances among sensors may result in various types of errors. For example, in field B, the map obtained with a 5.25 m distance between sensors (one sensor every seven crop rows) did not show relatively large weed patches (about 100 m^2^) present in this field; in field A, with high weed infestation throughout the field, significant errors were found in the map constructed with information every 5.25 m, showing weed patches much larger than those obtained in the map constructed with the detailed original data (maps not shown).

This optoelectronic mapping system could be combined with a patch spraying system using either a “mapping approach” or a “real-time” approach. In both cases, the results are relevant to determine the optimal size of management units (sub-units of the field to be sprayed). Berge *et al.* [23] estimated that 16% total errors (mapping plus spraying errors) could be expected by using standard 12-m boom sprayers and a 10-m image distance (minimum distance considering a vehicle speed of 7 km/h and a response time of 5 s). This spraying pattern corresponds to 120 m^2^ management units. These researchers found that, if acceptable error was restricted to 10%, the size of the management units should not exceed approximately 10 m^2^. For a 10-m boom and a response time of only 1 s, this would correspond to images taken every 1 m [23]. The optoelectronic mapping system tested in our study is able to record weed presence values every 0.3 m and, therefore, it could be used to spray units approximately three times smaller. However, considering that 1 s response time may be unrealistic, it would be preferable to work with spray booms with individually controllable segments with one sensor per segment. Using three optoelectronic sensors in a 12-m boom (the standard size in Spanish agriculture) or five sensors in a 20-m boom (more common in European agriculture) may be a cost effective procedure.

The optoelectronic system works well with different patterns of weed spatial distribution, with similar results in aggregated and not aggregated patterns. Previous studies conducted with discrete sampling methods showed that the reliability of sampling depends on the actual weed patch size and the spatial pattern of distribution of the species [34]. Similarly, simulation studies conducted by Berge *et al.* [23,37] have concluded that the suitability of patch spraying is likely to vary between fields due to differences in the spatial distribution of the weeds and their densities. If weed patches are elongated, well defined and wider than the sprayer boom, errors are likely to be smaller than when patches are very irregular in shape and narrower than the boom. Furthermore, if the average weed density is close to its threshold value in the field, it is likely to have relatively high spraying errors [37]. In contrast, the optoelectronic mapping system tested in this work provides a useful research tool to characterize the spatial structure of weed patches in a large variety of field conditions and to assess the suitability of various patch spraying patterns. Although the optoelectronic sensors are not able to discriminate between different weed species, this is not a major drawback in various types of situations: (a) when the herbicides to be used control a broad spectrum of weeds; (b) when herbicide treatments are planned to control only species that escaped pre-emergence treatments; (c) when total herbicides (e.g., glyphosate) are used on herbicide tolerant crops.

## 4. Conclusions

The optoelectronic mapping system tested in this study accurately produced weed maps in maize crops. The system verification, based on processing of digital images obtained in a regular grid, indicated that 83% of the weed presence was correctly located. A comparison among various sampling resolutions points out the possibility of obtaining precise weed maps using only one optoelectronic sensor every six crop rows. This would allow a significant reduction in equipment costs. Further tests in fields located at different growing areas as well as a validation of the mapping system in other crop rows (*i.e.*, vegetable crops) would be required to widen the use of this technique.

## Figures and Tables

**Table 1.** Contingency table showing the frequency of points with similar information on weed presence/absence (% agreement) when comparing the optoelectronic mapping system with digital images assessed by image processing sofware.

**Agreement (%)**	**Field A**	**Field B**	**Field C**	**Average**
Yes	78.1	81.4	88.4	82.6
No	21.9	18.6	11.6	17.4
% clean areas assessed as weedy	5.6	8.6	5.3	6.5
% weedy areas not detected	16.3	10.0	6.3	10.9

**Table 2.** Contingency table showing the frequency of points with similar information on weed presence/absence (% agreement) when comparing the reference maps constructed with optical sensors separated 0.75 m and the interpolated maps constructed with progressive increase of spacing between optical sensors. Asterisk (*) indicates that no spatial autocorrelation was found.

**Distance between sensors (m)**	**Field A**			**Field B**			**Field C**		
	**Agreement (%)**		**Not predicted^(1)^**	**Agreement (%)**		**Not predicted**	**Agreement (%)**		**Not predicted**
	**Yes**	**No**		**Yes**	**No^(1)^**		**Yes**	**No^(1)^**	
0.75 (control)	100	0	0	100	0	0	100	0	0
1.50	77	18	5	87	8	5	90	7	3
2.25	73	22	6	85	10	5	87	10	3
3.00	67	26	7	83	12	5	85	11	3
3.75	60	33	7	86	11	3	86	11	2
4.50	63	29	7	85	12	3	83	14	3
5.25	61	30	9	85	12	4	83	13	4
6.00	59	32	9	81	15	4	*	*	*

(1) Values from interpolated maps with a probability of 0.54 ≥ *P* ≥ 0.46 (see the Experimental Section).
